# Effect of Knee Orthosis Pressure Variation on Muscle Activities during Sit-to-Stand Motion in Patients with Knee Osteoarthritis

**DOI:** 10.3390/ijerph19031341

**Published:** 2022-01-25

**Authors:** Hiroaki Yamamoto, Shogo Okamatsu, Kodai Kitagawa, Chikamune Wada

**Affiliations:** 1 Department of Physical Therapy, Fukuoka Tenjin Medical Rehabilitation Academy, Fukuoka 810-0004, Japan; 2 Department of Life Science and Systems Engineering, Graduate School of Life Science and Systems Engineering, Kyushu Institute of Technology, Fukuoka 808-0196, Japan; rage_eye_enoki2@yahoo.co.jp (S.O.); kitagawakitagawa156@gmail.com (K.K.); wada@brain.kyutech.ac.jp (C.W.); 3 Department of Physical Therapy, Kitakyushu Rehabilitation College, Fukuoka 800-0343, Japan

**Keywords:** knee osteoarthritis, knee orthosis, wearing pressure, sit-to-stand motion, muscle activity

## Abstract

It is yet not known whether the variation in knee orthotics pressure would lead to changes in muscle activity during-sit-to-stand postural transition in patients with knee osteoarthritis (OA). Participants in this analytical study were patients with knee OA. The research design was a cross-sectional study. They were enrolled in the study through a sample of convenience method. The primary outcome measure was surface electromyography for measuring muscle activity while changing knee orthotics pressure during sit-to-stand motion. Data were summarized with mean and standard deviation while Friedman’s test was performed for multiple comparison of variables, at a significance level of *p* = 0.05. Seven elderly patients with knee osteoarthritis (mean age 71.4 ± 11.8 years) participated in the study. Moderate orthotics (7.3 mmHg) led to a significant increase in the percentage maximum voluntary contraction (MVC) of tibialis anterior compared to that obtained without orthotics. Rectus femoris, vastus medialis, vastus lateralis, and biceps femoris tended to increase the % MVC with an increase in wearing pressure. It was therefore concluded that the muscle activity during sit-to-stand motion could be increased in patients with knee osteoarthritis by wearing flexible orthotics with varying pressure.

## 1. Introduction

Currently, 28% of the population in Japan is aged 65 years old or over [1]. The increase in the aged population is often accompanied by high prevalence rate of age-related degenerative diseases such as knee osteoarthritis (OA). In a large-scale inhabitant cohort ROAD (Research on Osteoarthritis/Osteoarthrosis Against Disability) project conducted in 2005, the prevalence of knee OA was 54.6% (42.0% for male and 61.5% for female) [2]. This study was conducted in Japan with 3040 participants in the survey. The radiographic degree or presence was diagnosed using X-ray images of 3040 participants in the baseline survey of the ROAD study. That is, a standing X-ray image is taken. The KL classification (Kellgren–Lawrence grading system), which is a severity classification based on the X-ray image of knee OA, is classified by an orthopedic surgeon, and the KL classification is performed on at least one knee joint of the left and right knees. When Grade 2 or higher was diagnosed in the knee osteoarthritis, it was regarded as knee OA.

Knee OA causes movement-related pain and dysfunction, impairs activities of daily living (ADL), and significantly reduces the quality of life (QOL) in aged individuals [1]. When the ability to control movements is impaired in the knee joint, patients with knee OA are more susceptible to anterior shear force, rotational stress, and compression stress on the worn patellofemoral joint surface [3]. This pathomechanical process often leads to pain occurrence in the knee during sit-to-stand postural transitions, especially in ADL. Sit-to-stand movement is frequently repeated in daily life and is considered difficult in elderly people with muscle weakness, and patients with locomotor disorders in the lower limbs [4]. Clinically, difficulty in the performance of sit-to-stand movement may adversely affect ADL.

The knee orthosis is an effective conservative intervention to improve the quality of life [5]. Therefore, flexible knee orthosis is prescribed for patients with knee OA in clinical practice to reduce movement-related pain, [6] and to improve balance function [7]. Knee orthoses have been documented to influence proprioceptors and physical functions by compressing and stimulating the muscles around the knee joint [8]. Improving intrinsic sensation in patients with osteoarthritis of the knee can reduce pain [9]. Additionally, the use of flexible knee orthosis exerts pressure on the knee joint, thereby producing a sense of security, which is thought to improve balance function. Moreover, both two types of flexible knee orthosis—one covers the patella and the other does not—are effective [10].

Although the therapeutic effect of flexible knee orthosis has been reported, the effect of variation in the pressure is not sufficiently documented. Knee orthosis is therapeutically applied to correct and prevent deformity, control and fix joint movements, and assist joint movements [11]. It is suggested that increasing the wearing pressure of the knee OA fixes the knee joint, facilitates joint movement, and activates muscle activity. Therefore, in this study, we investigated whether muscle activity during sit-to-stand motion of the knee would be affected by variations in orthosis wearing pressure. We hypothesize that increasing wearing pressure of flexible knee orthotics would change muscular activities around the knee joint during sit-to-stand motion in patients with knee OA.

## 2. Materials and Methods

### 2.1. Participants

The participants in this study were seven patients with knee OA. Seven patients were randomly selected based on the exclusion criteria from the patients with knee osteoarthritis attending outpatient clinics. The exclusion criteria are that the patient has cerebrovascular disease, neurological disease, dementia, or a history of lower limb orthopedic surgery other than knee OA. The severity of knee OA was determined using the KL classification as an index [12], with 3 patients in stage II, 3 patients in stage III, and 1 patient in stage IV. Additionally, those with a history of lower limb orthopedic surgery other than cerebrovascular accident, neurological disease, dementia, and knee OA, were excluded. The research design was a cross-sectional study.

### 2.2. Instrumentation/Materials for Data Collection

**Figure 1:**
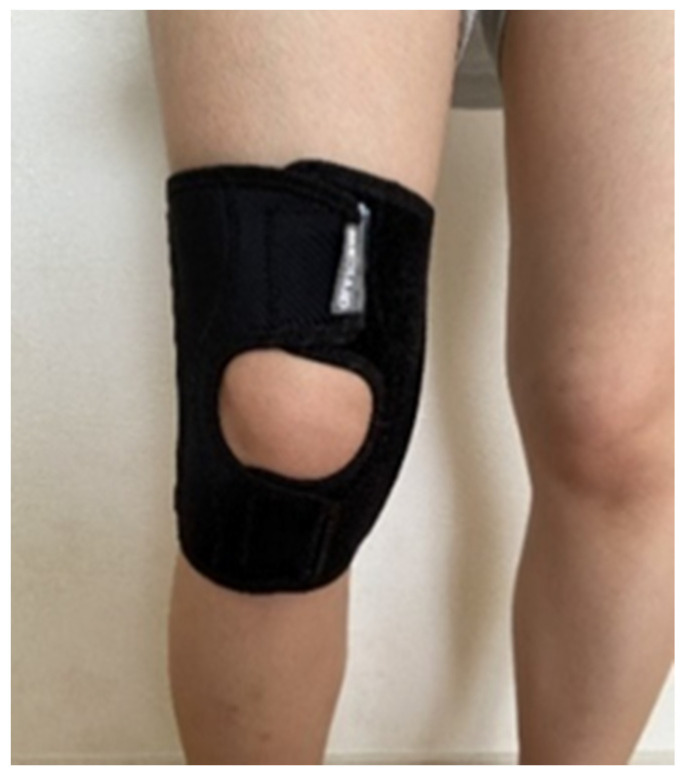
Flexible knee orthosis.

An open-type flexible knee orthosis (Nippon Sigma Max Co, Ltd., Shinjuku-ku, Japan) was used in this study (Figure 1). The wearing pressure of the orthotics was measured using an airbag-type pressure sensor “Palm Q” (manufactured by Cape, Ltd., Osaka, Japan). Japan Knee Osteoarthritis Measure (JKOM) (Table 1) is a disease-specific quality of life (QOL) evaluation scale for knee OA. It was used to assess the degree of pain and stiffness as well as the daily living functions, general activity, and health condition rated on 5-likert scale ranging from 1 (no pain, no difficulty), indicating the best condition, to 5 (severe pain, difficult), indicating the worst condition. Femoral-tibial alignment (FTA) was adopted to assess the degree of genu varum in the knee joint. The classification of the severity of knee OA was determined using Kellgren–Lawrence (K–L) classification grading system as an index [13]. The percentage maximum voluntary contraction (% MVC) was measured using surface electrodes to estimate muscular activities.

### 2.3. Procedure

This study was conducted in compliance with the Declaration of Helsinki and was approved by the Ethics Committee of Fukuoka Tenjin Medical Rehabilitation Academy (approval number 2020–1). The purpose and method of this study were explained to the patients, after which their written consent was sought and obtained to participate in the study.

### 2.4. Assessment of Tibio-Femoral Alignment

**Figure 2:**
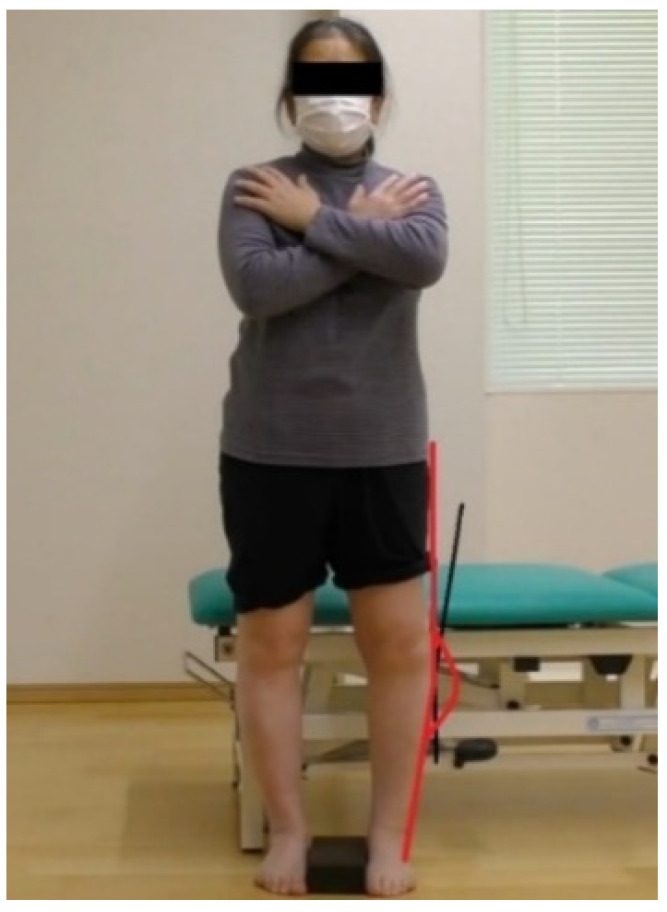
Measurement of Femoral-tibial alignment by body surface image.

The camera was placed 1.0 m away from the subject so that the markers on the body landmarks could be photographed. The patients were photographed in a standing posture with a 0.1 meter-wide plate placed between their feet, in order to be able to detect the marker in the photograph taken. Thereafter, the intersection of the straight line connecting the greater trochanter and the marker attached to the lateral condyle of the femur, and that connecting the peroneal head and the marker attached to the lateral ankle was calculated (Figure 1), using the image processing software ImageJ (National Institutes of Health). The outer angle calculated from the body surface image has been reported to be strongly correlated with the femoral-tibial alignment (FTA) assessed with plain film radiograph (X-ray image) [17]. This (anatomical) method was preferable as patients were measured without exposure to X-ray (Figure 2).

### 2.5. Severity of Osteoarthritis and Quality-of-Life Assessment

Quality of life (QOL) of the patients was evaluated by checking the degree of pain over the last few days with the visual analog scale component of JKOM, in which the patients were asked to point to their degree of pain on a 10 cm line that ranges from “no pain” to “the most severe pain I have ever experienced.” This was followed by the assessment of patients’ pain and stiffness aspect of the tool.

### 2.6. Sit-to-Stand Motion Assessment

**Figure 3:**
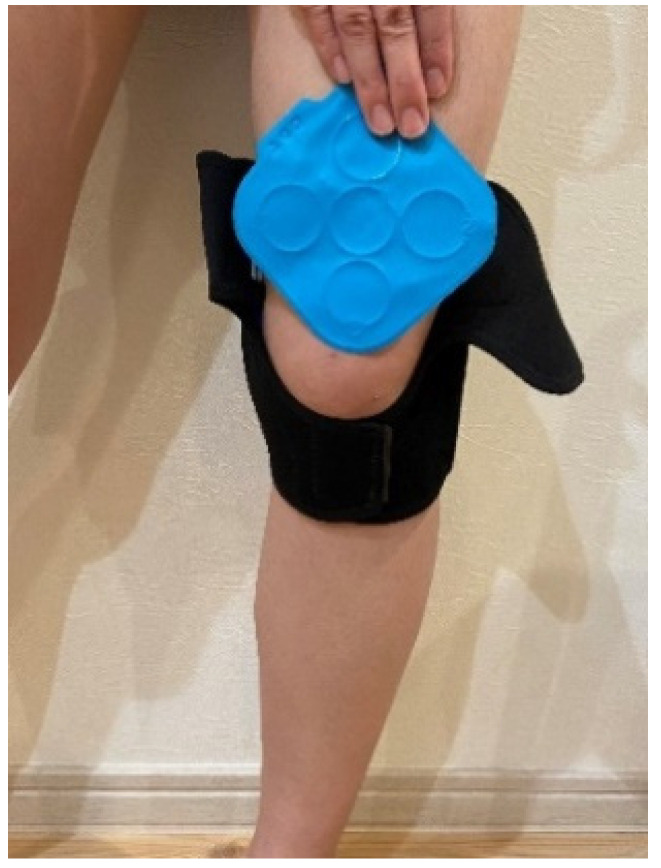
Palm Q sticking position.

The sit-to-stand movement was performed with the patient assuming an initial erect posture to ensure vertical lower limb position. Additionally, the feet were placed in such a way that the distance between both feet was equal to the distance between both acromion. The size of the orthotics was decided so that the perimeter of the thigh at 10 cm above the patella center in the affected side was close to the median of the coverage range of the orthotics. The three wearing pressure patterns of orthotics were then prepared: (1) Control (without the orthotics), (2) Low Pressure (orthotics loosely worn so that it would not slip off), (3) Moderate Pressure (orthotics worn at an appropriate pressure), and (4) High Pressure (the hook-and-loop fastener of the orthotics was tightened to the maximum). According to Laplace’s law [18], the wearing pressure exerted on the lower limb is directly proportional to the tension generated from the orthotics applied to the curved surface of the lower limb. The center of the pressure sensor (10 cm^2^) was set to be 5 cm above the center of the patella, and the orthosis was attached on the sensor (Figure 3).

Patients were then asked to sit in such a way that the center of his/her thigh corresponded to the edge of the chair as the initial posture. They were asked to rise to a standing position at their own pace without shifting their lower limbs. Patients were made to perform a trial movement once before the actual measurement.

### 2.7. Measurement of Muscular Activities

**Figure 4:**
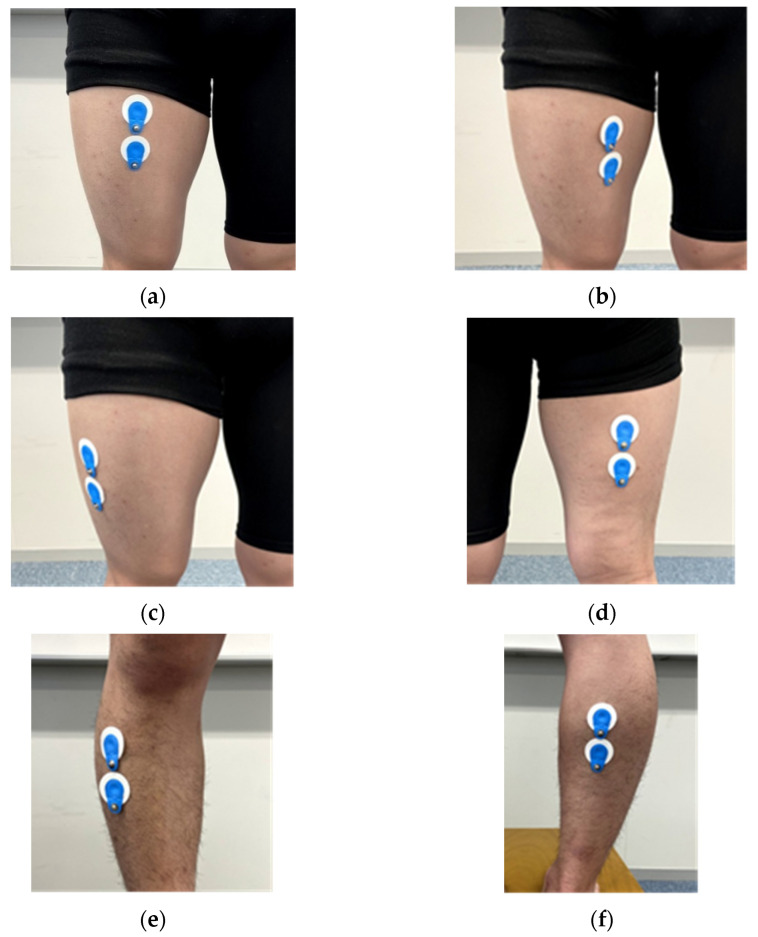
Pasting position of surface electromyography (**a**) rectus femoris, (**b**) the vastus medialis, (**c**) vastus lateralis, (**d**) biceps femoris, (**e**) tibialis anterior, and (**f**) gastrocnemius.

The percentage MVC of the rectus femoris, (RF), vastus medialis (VM), vastus lateralis (VL), biceps femoris (BF), anterior tibialis anterior (TA), and gastrocnemius (GC) were measured. Two electrodes were placed on each muscle (with 3 cm spacing) so that they were parallel with the muscle fibers (Figure 4). The landmarks for the electrodes attachments for each target muscle are as follows; (1) RF: the midpoint of the line connecting the anterior superior iliac spine and the upper edge of the patella [19], (2) VM: the point 80% distal on the line connecting the anterior superior iliac spine and the knee joint space [20], (3) VL: the point of 1/3 distal on the line connecting the greater trochanter and the lateral epicondyle of the femur [20], (4) BF: the midpoint of the line connecting the fibula head and the ischial tuberosity [19], (5) TA: the point connecting the four lateral fingers distal from the tibial tuberosity and one-lateral-finger lateral from the tibial crest [19], and (6) GC: the point connecting five-lateral-fingers distal to the popliteal skin line and lateral to the posterior surface of the lower leg [19]. After rectification, the percentage MVC data were obtained for each electromyography by normalizing the scores with the maximum isometric contraction. The assessment of maximum isometric contraction of RF, VM, VL, and TA, for the manual muscle strength test, had been carried out in the sitting position before the patient stood up [21]. Although the measurement of maximum isometric contraction for BF and GC had been reported in lying or standing position, in this study, we used different measurement methods in a sitting position for BF and GC. For the BF, the trunk electrode was placed in the median position to the hip joint in the sitting position and the knee joint was flexed at 45 degrees in the middle position of the hip joint. In measuring the maximum isometric contraction, the assessor applied force to the distal posterior surface of the lower leg to avoid hip flexion. For GC measurement, the knee joint was in a slight flexion in the sitting position, with ankle plantar flexion at 45°. The assessor applied force to the sole of the foot to avoid knee extension. The integral value of percentage MVC normalized by the operating time was used as an evaluation index of muscle activity. The operating time was from the starting point of the standing-from-sitting process, to one second after standing posture.

### 2.8. Data Analysis

Statistical analysis was performed using STAT Windows statistical software. Sociodemographic data were presented as mean, standard deviation, and percentage. Multiple comparisons of muscle activity during sit-to-stand motion among the Control, Low Pressure, Middle Pressure, and High Pressure was performed using Friedman’s test and Tukey’s method. The significance level was set at 5%.

## 3. Results

### 3.1. Sociodemographic Characteristics of the Patients

Seven elderly patients were enrolled into this study, made up of six males and one female. The mean age, height, weight, and BMI of the participants were 71.4 ± 11.8 years, 154.8 ± 10.9 cm, 59.0 ± 15.2 kg, and 24.2 ± 3.6, respectively. Three patients apiece presented with stage II, and stage III of the Kellgren–Lawrence grading system while one patient was in stage IV.

### 3.2. The Degree of the Severity of Knee OA

The femoral-tibial alignment (FTA) and JKOM scores for each subject are shown in Table 2. The minimum femoral-tibial alignment (FTA) angle was 181.4 degrees, while the maximum angle was 194 degrees, and the mean was 188.1 ± 4.3 degrees. All subjects had genu varum. The minimum score of JKOM was 6, and the maximum score was 48 points, giving a mean of 24.3 ± 12.3 points.

### 3.3. The Variation in the Knee Orthotics Pressure and Muscle Activities

The wearing pressures were 1.6 mmHg, 7.3 mmHg and 18.1 mmHg for the Low Pressure, Moderate Pressure, and High Pressure conditions, respectively.

A comparison of each muscle and each wearing pressure are shown in Table 3. In the TA, muscle activity was significantly reduced in Middle pressure compared with that of the Control condition. Additionally, the four muscles of the thigh (RF, VM, VL, BF) tended to increase muscle activity when the wearing pressure of knee orthosis was increased. The GC muscle did not differ significantly in all conditions (Table 3).

## 4. Discussion

The primary focus of this study was to investigate the effect of knee orthotics wearing pressure on the muscle activity during sit-to-stand postural transition in patients with knee osteoarthritis. We found that the TA activity was significantly reduced with Moderate orthotics wearing pressure compared with that of the control. Additionally, the muscle activity of the thigh tended to increase when the wearing pressure of the knee orthosis was increased. These results are considered to show the possibility of easiness in standing up by suppressing overactivity of the TA muscle and promoting efficient muscle activity in the thigh.

The remarkable reduction in the activity of TA could be attributed to the fixed position of the knee joint while wearing orthotics. The angle range of knee joint flexion during sit-to-stand is about 100 degrees [22]. If the angle range in both of knee joint and ankle joint is inadequate, the center of gravity will not shift sufficiently [23]. It is considered that the fixation of the knee joint increased the angle range of knee joint and ankle joint during the sit-to-stand motion and the center of gravity could be moved forward efficiently. Generally, it has been reported that the TA contracts maximally when the lower leg is tilted forward in the sit-to-stand motion, and the TA plays an important role in the postural transition [10]. Orthotics is commonly prescribed to provide support against the instability and abnormal movement caused by the disorder of the joint component. Additionally, orthotics reproduce physiological movement of the joints to prevent further impairment in the joint components [24]. To reduce the compressive force on osteoarthritic knee joint during sit-to-stand motion, the muscles around the knee joint must work efficiently to withstand the initial shear stress [25]. Furthermore, the knee is stabilized largely by the activity of the VM at the beginning of the sit-to-stand postural change [26], thus it presupposes that the knee joint was stabilized by the patient wearing the knee orthotics and the knee joint was flexed efficiently by moving the center of gravity anteriorly.

Since the sole of the foot was in contact with the floor, dorsiflexion of the ankle occurred in a closed chain with the flexion of the knee joint during the sit-to-stand motion. Without knee orthotics, the TA became overactivated to compensate the insufficiency of knee flexion angle and ankle dorsiflexion angle. However, under the Moderate wearing pressure, knee flexion, and ankle dorsiflexion angle increased as the center of gravity moved anteriorly, thus providing an efficient postural transition. As a result, it was assumed that the overactivity of the TA was suppressed under this condition.

Additionally, the muscle activity of the thigh tended to increase when the wearing pressure of the knee orthosis increased. This occurrence could be adduced to the stimulation of muscle fiber activity due to pressurization. It has been reported that increased muscle strength and muscle hypertrophy occur when blood flow is restricted due to pressurization, even with sub-optimal load [27]. In a study comparing muscle output from the use of knee orthotics and taping, the output of the knee joint muscle increased as the wearing pressure orthosis increased [28]. The orthosis and taping were considered to play an auxiliary role in compensating for the lack of muscle activity during movement, thereby facilitating the movement [28]. Our findings agreed with the previous study. The knee orthotics used in this study covered the knee joint and its perimeter, so it did not directly pressurize the lower leg. Therefore, it is considered that four of the six target muscles in the thigh were affected by pressurization caused by the orthotics. However, co-contraction of the medial knee muscles may contribute to the progression of knee OA. Therefore, altering the pattern of muscle activity around the knee may slow the progression [29].

The number of participants in this study was not large and this was a limitation, but most patients were grade 2 or 3 and had moderate knee OA. Therefore, the results were considered to be definitively applied for the patients with moderate knee OA. However, more investigation is necessary to show that the results could apply for the mild or severe (KL classification I or IV). In addition, “sit-to-stand” was focused on in this study because there were patients with osteoarthritis of the knee who have difficulty in standing up. The results show that knee braces were able to facilitate lifting the body when the wearing pressure was increased, and then it is considered there is a possibility of improving ADL and QOL. However, investigation during standing and walking motion should be performed to improve the ADL and QOL. For the next research study, we plan to increase the number of participants and investigate other motions. 

## 5. Conclusions

It was concluded that increasing the wearing pressure of knee orthotics could lead to increased muscle activity around the knee joints. Thus, adequate regulation of flexible knee orthotics pressure could be beneficial for patients with knee OA during postural transition into standing posture. In the future, we will proceed with the development of flexible knee orthoses focusing on wearing pressure.

## Figures and Tables

**Table 1:** Japan Knee Osteoarthritis Measure.

Pain and stiffness in knees
1. Do you feel stiffness in your knees when you wake up in the morning? 2. Do you feel pain in your knees when you wake up in the morning? 3. How often do you wake up in the night because of pain in your knees? 4. Do you have pain in your knees when you walk on a flat surface? 5. Do you have pain in your knees when ascending stairs? 6. Do you have pain in your knees when desending stairs? 7. Do you have pain in your knees when bending to the floor or standing up? 8. Do you have pain in your knees when standding?
II. Condition in daily life
9. How diffcult is ascending or descending stairs? 10. How diffcult is bending to the floor or standing up? 11. How diffcult is standing up from sitting on a westem style toilet? 12. How diffcult is wearing pants, skirts, and underwear? 13. How diffcult is putting on socks? 14. How long can you walk on a flat surface without taking a rest? 15. Have you been using a walking stick (cane) recently? 16. How diffcult is shopping for daily necessities? 17. How diffcult is doing light housework (cleaning the dining room after eating, etc)? 18. How diffcult is doing heavy housework (using the vaccum cleaner, etc)?
III. General activities
19. Have you gone to an event or to a department store during the last month? 20. Were things that you usualy do (some kind of leason, meeting friends, etc) diffcultbecause of knee pain during the last one month? 21. Did you limit doing things you usualy do because of knee pain during the last month? 22. Did you despair of going outside somewhere close because of knee pain during the last month? 23. Did you despair of going outside somewhere far because of knee pain during the last month?
IV. Health condisions
24. Do you think your health during the last month is average? 25. Do you think that knee pain has been negatively affecting your health during the last month?

Item 1–8 cited from [14]. Item 9–14 cited from [15]. Item 21–25 cited from [16].

**Table 2:** Femoral-tibial alignment (FTA) and JKOM (list of subjects).

	FTA	JKOM
ID1	189.8	14
ID2	181.4	6
ID3	188.8	28
ID4	182.7	27
ID5	193.1	19
ID6	192.4	28
ID7	187.3	48
Mean ± SD	188.1 ± 4.3	24.3 ± 12.3

Femoral-tibial alignment (FTA) unit: degrees. JKOM unit: points.

**Table 3:** Muscle activity during standing motion.

	Control	Low Pressure	Middle Pressure	High Pressure
Rectus femoris	33.8 ± 18.4	22.9 ± 15.4	27.8 ± 19.5	33.3 ± 20.4
Vastus medialis	38.0 ± 20.6	27.0 ± 11.1	28.6 ± 15.0	37.5 ± 17.9
Vastus lateralis	41.9 ± 15.5	29.6 ± 13.3	35.7 ± 18.8	41.5 ± 14.2
Biceps femoris	34.8 ± 10.6	29.7 ± 16.7	35.7 ± 28.2	36.1 ± 21.7
Tibialis anterior	25.5 ± 17.9	19.3 ± 9.2	16.4 ± 8.8	23.6 ± 16.7
Gastrocnemius	47.2 ± 16.8	44.1 ± 23.9	44.3 ± 23.7	49.4 ± 25.4

Unit: % MVC.

## Data Availability

Data are stored in a password-protected PC located in the Kyushu Institute of Technology.
